# Regulatory T cells inhibit FoxP3 to increase the population of tumor initiating cells in hepatocellular carcinoma

**DOI:** 10.1007/s00432-024-05892-2

**Published:** 2024-07-29

**Authors:** Chang Liu, Yi-jun Tu, Hong-Yang Cai, Yan-yan Pan, Yuan-yuan Wu, Li Zhang

**Affiliations:** Central Hospital of Dalian University of Technology, No. 826, Southwest Road, Dalian, 116033 China

**Keywords:** Tumor initiating cells, Cancer stem cells, Regulatory T cells, FoxP3

## Abstract

**Purpose:**

Tumor initiating cells (TICs) or cancer stem cells (CSCs) are considered to be the main culprit of hepatocellular carcinoma (HCC) initiation and progression, nevertheless the mechanism by which tumor microenvironment maintains the HCC ‘stemness’ is not fully understood. This study aims to investigate the effect of regulatory T cells (Tregs) on the TICs characteristics of HCC.

**Methods:**

Immunocytochemistry, flow cytometry, real-time PCR, western blot, in vitro sphere-formation, and in vivo tumorigenesis assay were used to detect HCC ‘stemness’. Additionally, after forced expression or inhibition of FoxP3, β-catenin expression and HCC ‘stemness’ were investigated.

**Results:**

Tregs enhanced the ‘stemness’ of HCC cells by upregulating TIC-related markers CD133, Oct3/4, Sox2, c-Myc, Klf4, Nanog, CD13, EpCAM, and inducting epithelial to mesenchymal transition (EMT), increasing TICs ratio, as well as promoting tumorigenic ability. Moreover, β-catenin and c-Myc were upregulated in HCC cells after co-cultured with Tregs. HCC ‘stemness’ was inhibited after treatment with Wnt/β-catenin pathway inhibitor. Furthermore, forced expression of FoxP3 resulted in increased GSK3β, decreased β-catenin and TIC ratio in HCC. In contrast, FoxP3 interference reduced GSK3β, enhanced β-catenin and TIC ratio of HCC.

**Conclusion:**

This study, for the first time, demonstrated that Tregs increased the population of TICs in HCC by inhibiting FoxP3 as well as promoting β-catenin expression.

**Supplementary Information:**

The online version contains supplementary material available at 10.1007/s00432-024-05892-2.

## Introduction

HCC is the most prevalent primary liver cancer, which ranks as the sixth most common cancer and the third most leading cause of cancer-related death worldwide. A higher incidence rate of hepatitis B virus-related HCC occurs in China, and over 50% of HCC-related deaths are in China (Sung et al. 2021). Late diagnosis, frequent relapse, and the refractory nature to chemotherapy render HCC an intractable disease.

Due to the inherently high genetic instability, a small population within the HCC has evolved with the ability of to initiate and maintain cancer growth. Rapidly growing evidence has demonstrated that some HCCs, if not all, are caused by the activation of TICs. Their resistance to anticancer drugs has been an obstacle for the total eradication of HCC (Lai et al. 2019). TICs or CSCs are defined as a population of cells found within a tumor that have characteristics similar to normal stem cells. The extraordinary capacities of self-renewal, tumorigenicity, and differentiation endow TICs with a pivotal role in tumor relapse, therapy resistance, and metastasis. HCC TICs or CSCs can develop from mature hepatocytes, hepatoblasts, and biliary cells due to liver damage, regeneration, or oncogenic dedifferentiation. TICs can originate from stem cell transformation or dedifferentiation of progenitor cells (Quiroz Reyes et al. 2023). CD133 was first suggested as a particular HCC TIC marker. Then, a number of molecular markers have been identified, such as CD90, EpCAM, CD24, CD13, SOX9, ABCG2, CD44, ALDH, and side population (SP) (Lee et al. 2022). TICs typically harbor persistent activation of highly conserved ‘stemness’-related signaling pathways, including Wnt/β-catenin, Hedgehog, and Notch. Wnt/β-catenin signaling has been reported to be one of the most active signaling pathways indispensable to self-renewal and drug resistance of HCC TICs (Kang et al. 2019).

Tregs are a subset of CD4^+^ CD25^+^ CD127^−^ T lymphocytes that constitutively express the transcription factor FoxP3 (forkhead box P3). Activated Tregs inhibit different subsets of immune cells via contact-dependent ways between checkpoint molecules and their ligands involving PD-1, PD-L1, CTLA-4, GITR, Tim-3, and galectin-9 (Langhans et al. 2019). Treg number has been shown to increase with advanced tumor stage and correlate with poor prognosis of HCC (Fu et al. 2007; Wang et al. 2016).

It was found that Foxp3^+^ Treg cells were capable of inducing colorectal cancer cells to become cancer-initiating cells (Yang et al. 2011). Additionally, Tregs could induce the expression of core cancer stem cell-related genes and spheres formation adbility, resulting in increased cancer stemness and tumorigenic potential of glioma cancer cell (Liu et al. 2021). Nevertheless, the role of Tregs, which reside in the HCC tumor environment, in the regulation of HCC cellular behavior, including proliferation, metastasis, and especially TICs characteristics, was undetermined. In this study, it was found for the first time that Tregs enhanced the ‘stemness’ of HCC by inhibiting FoxP3 and up-regulating β-catenin.

## Materials and methods

### Materials

All chemicals were purchased from Sigma-Aldrich (St. Louis, MO, USA) unless otherwise specified. Sodium alginate (Qingdao Jingyan Bio-Tech, Qingdao, China) was purified by removing protein and endotoxin, according to the protocol used in our laboratory. XAV-939 was purchased from MedChemExpress (Monmouth Junction, NJ, USA).

### Human sample

The use of human subjects was reviewed and approved by the Ethics Committees of Dalian Municipal Central Hospital and 2nd Clinical Medical College of Jinan University, and all work was conducted in accordance with the Declaration of Helsinki (1964). The experiment was conducted with the human subjects’ understanding and consent.

20 mL of peripheral blood was obtained from a 41-year-old male patient with advanced HCC. The serum was placed in a 56 ℃ water bath for 30 min for inactivation. The mononuclear cells were then isolated by gradient centrifugation with lymphocyte separation medium (Lymphoprep, 08751, STEMCELL Technology, Vancouver, BC, Canada).

### Treg cell isolation and expansion

Tregs were isolated from peripheral blood mononuclear cells of the HCC patient using the CD4^+^CD25^+^CD127^dim/−^ Regulatory T Cell Isolation Kit (130-094-775, Miltenyi Biotec, Bergisch Gladbach, Germany). Briefly, the isolation of CD4^+^CD25^+^CD127^dim/–^ regulatory T cells was performed with a cocktail of biotinylated antibodies and anti-Biotin microbeads for the depletion of non-CD4^+^ and CD127^high^ cells. Then, the flow-through fraction of pre-enriched CD4^+^ CD127^dim/–^ T cells is labeled with CD25 microbeads for subsequent positive selection of CD4^+^ CD25^+^ CD127d^im/–^ Treg cells using MidiMACS™ Separator and Starting Kits (130-042-301, Miltenyi Biotec).

Tregs were expanded in X-VIVO™ 15 medium (BE02-060 F, Lonza, Basel, Switzerland) supplemented with 2% heat-inactivated patient serum, 500 U/mL recombinant human IL-2 (T&L Biological Technology, Beijing, China), MACSiBeads pre-loaded with CD3 and CD28 antibodies (130-095-353, Miltenyi Biotec), and 10 ng/mL rapamycin (HY-10,219, MedChemExpress).

### HCC cell culture, encapsulation

Human HCC cell line HCC-LM3, purchased from Cellcook (Cellcook Biotech, Guangzhou, China), has recently been authenticated by karyotype analysis. HCC-LM3 cells were cultured in high glucose Dulbecco’s Modified Eagle’s Medium (H-DMEM, Gibco, Carlsbad, CA, USA) supplemented with 10% Fetal Bovine Serum (FBS, Gibco) in a 37 °C incubator with an atmosphere of 5% CO_2_.

Single cells dissociated from monolayer cultures were counted and suspended in 2.5%, (w/v) sodium alginate at a cell density of 1 × 10^6^/ml. The cell suspension was extruded into 100 mM CaCl_2_ solution. The gelation time to produce calcium alginate gel (ALG) beads was 30 min.

### HCC cells and Tregs co-culture

HCC-LM3 cells formed tumor spheres in ALG beads after 10 days of culture. Then the ALG beads encapsulated HCC-LM3 cells were co-cultured with Tregs for 3 days in H-DMEM supplemented with 10% FBS in a 37 °C incubator with an atmosphere of 5% CO_2_, the ratio of HCC cells and Tregs were 10:1. Tregs and HCC cells were separated by sedimentation and filtration with 100 μm strainer to remove Tregs (352,360, Corning, NY, USA). The encapsulated HCC cells were harvested from ALG beads by treating with 55 mM sodium citrate, and then used for further experiments.

### Plasmid, shRNAs and cell transfection

The plasmids for generating vectors were prepared from p-CMV-GreenZeo (Genechem, Shanghai, China). Short-hairpin small interfering RNA sequences were 5’-GAAGCAGCGGACACTCAAT-3’, 5’-ACACGCATGTTTGCCTTCT-3’, and 5’-TGGCAAATGGTGTCTGCAA-3’. A scrambled sequence (5’-TGACGCGATACGTATTGTA-3’) was used as a negative control. Transfection of HCC-LM3 cells were performed using Lipofectamine 2000 (Invitrogen, Carlsbad, CA, USA). DNA-liposome complexes were prepared at 4˚C to a final volume of 1 µg/µl and added to HCC-LM3 cells (1 µg/ml). Transfection was performed for 6 h at 37 ℃.

### Flow cytometry

Tregs were labeled with FITC Mouse Anti-Human CD4 (1:5) (561,005, BD Biosciences, Franklin Lakes, NJ, USA), PE Mouse Anti-Human CD25 (1:5) (555,432, BD Biosciences) and Alexa Fluor^®^ 647 Mouse anti-Human FoxP3 (1:20) (561,184, BD Biosciences) antibodies for 30 min on ice, followed by washing with phosphate buffered saline (PBS) (Gibco), FITC Mouse IgG1 (555,748, BD Biosciences), PE Mouse IgG1 (555,749, BD Biosciences), Alexa Fluor^®^ 647 Mouse IgG1 (557,732, BD Biosciences) were used as isotype controls. As for FoxP3 staining, Human FoxP3 Buffer Set (560,098, BD Biosciences) was used. Briefly, Tregs were fixed with Buffer A, incubated for 10 min at room temperature (RT), and permeabilized with buffer C, incubated for 30 min at RT. Flow cytometry was performed by FACSCanto II flow cytometer (BD Biosciences), and the data were analyzed and presented using Flowjo software version 10 (Flowjo, Ashland, OR, USA).

### Quantitative reverse transcription polymerase chain reaction (RT-qPCR)

RT-qPCR (two-step method) was applied to examine the relative levels of the genes, using GAPDH as an internal control. The total RNA was isolated using TRIzol^®^ reagent (Invitrogen), according to the manufacturer’s instructions. Reverse transcription (RT) was performed using a PrimeScript RT Reagent Kit (RR036A, TaKaRa, Shiga, Japan). Real-time PCR was carried out with SYBR Premix Ex Taq (Perfect Real Time) (RR820A, Takara). PCR amplification and fluorescence detection were performed using a LightCycler^®^ 96 System (Roche, Basel, Swiss). The primers used in this study were listed in supplementary Table 1. The results were presented as the calculated comparative expression ratios of the target sample to the control group for each sample using the Ct method (2^−∆∆ Ct^).

### Immunofluorescence staining

HCC-LM3 cell spheres were fixed with 4% paraformaldehyde (PFA) and washed with PBS (Gibco) three times. After cytospin preparation, cells were treated with 0.05% Triton-X 100 (Sigma-Aldrich), then incubated with CD133 primary antibody (1:400) (64,326, Cell Signaling Technology, Danvers, MA, USA) in PBS containing 1% goat serum (16,210,064, Thermal Fisher Scientific, Waltham, MA) at 4 °C overnight. Then the cells were treated with Alexa Fluor^®^ 555 conjugated anti-rabbit IgG antibody (1:1000) (4413, Cell Signaling Technology) for 60 min at RT. The primary antibody was omitted for negative control. Nuclear staining was performed using Hoechst 33,342 (H3570, Thermal Fisher Scientific). The samples were observed using an inverted fluorescence microscope (DMI8, Leica, Solms, Germany).

### Western blot

Cells were lysed in a lysis buffer containing protease and phosphatase inhibitors (Keygentec, Nanjing, China). Protein concentration was quantified by the BCA protein assay kit (Keygentec), and an equal amount of protein was loaded in each lane. Constant voltage electrophoresis was carried out with 10% polyacrylamide gels. Then the proteins were transferred to polyvinylidene fluoride (PVDF) membranes (Merk, Massachusetts, USA). The PVDF membranes were blocked with 3% bovine serum albumin (BSA) (Sigma) and hybridized with anti-c-Myc antibody (1:100) (sc-373,712, Santa Cruz, Dallas, TX, USA), anti-FoxP3 antibody (1:200) (sc-166,212, Santa Cruz), anti-GSK3β antibody (1:200) (sc-71,186, Santa Cruz), anti-β-catenin antibody (1:200) (sc-7963, Santa Cruz), and anti-β-actin antibody (1:500) (sc-47,778, Santa Cruz) overnight at 4 ˚C. After washing with TBST (Tris-buffered saline and Tween 20) (Keygentec), the PVDF membranes were incubated with secondary antibodies (PV9000, ZSGB-bio, Beijing, China) at room temperature for 60 min, followed by washing with TBST. Finally, PVDF membranes were covered with 3, 3-Diaminobenzidine (DAB) (ZLI-9017, ZSGB-bio) for the display of specific protein bands.

### Sphere formation assay

HCC-LM3 cells from control and co-culture groups were trypsinized into single cells and resuspended in CSCs medium consisting of DMEM/F-12 (Invitrogen) supplemented with epidermal growth factor (PHG0311, Gibco), basic fibroblast growth factor (PHG0266, Gibco), insulin (41,400,045, Gibco), B27 (17,504,044, Gibco). The cells were seeded at a density of 1 × 10^4^ cells/well in ultra-low attachment 6-well plates. After 21 days of culture with replenishment of one-half of the medium every 3 days, tumor spheres were observed and counted.

### In vivo tumorigenesis assay

All animal experiments were approved by the Jinan University Laboratory Animal Ethics Committee. Male BALB/c nude mice, 4–6 weeks of age, were used in this study. 5 × 10^6^ HCC-LM3 cells harvested from ALG beads before and after co-cultured with Tregs were suspended in 100 µl saline supplement with 50% Matrigel (BD Biosciences), respectively, and then injected subcutaneously into the dorsal flanks of mice. Each experimental group included six mice. Animals were sacrificed after 6 weeks, and tumor volume (cm^3^) was measured weekly using electronic calipers and calculated with the formula (length × width × height) × Π/2.

### Statistical analysis

All individual in vitro experiments were performed with at least three replicates. Data were expressed as means ± standard deviation (SD). The significance of differences between the two groups was determined using unpaired Student’s t-tests. Differences were considered significant at *P* < 0.05.

## Results

### Tregs isolation and characterization

**Fig. 1:**
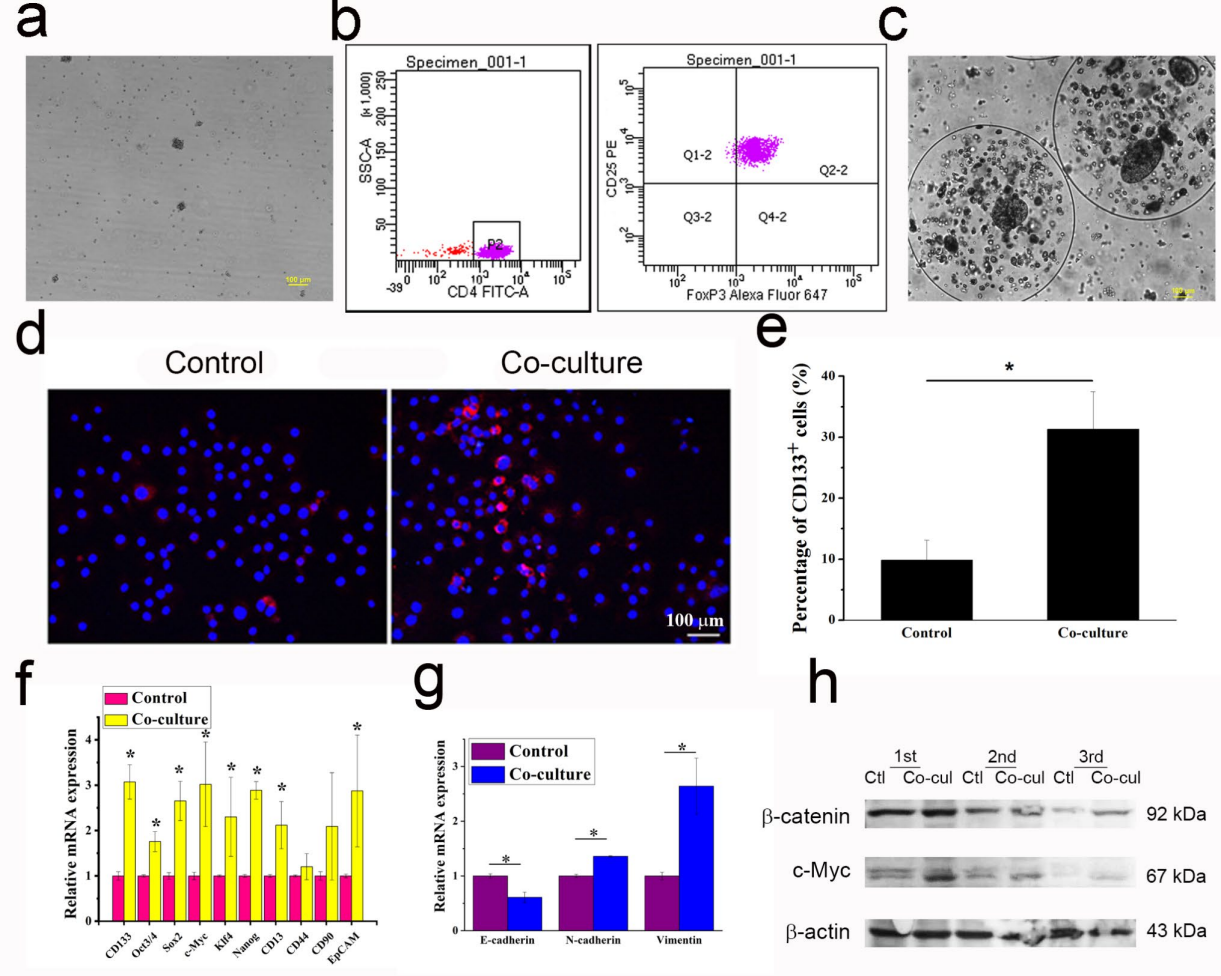
‘Stemness’ of HCC-LM3 cells was enhanced after co-cultured with Tregs. (**a**) Isolation and expansion of CD4 + CD25 + CD127- Tregs. (**b**) Flow cytometry analysis showing high expression of CD4, CD25, and FoxP3 in Tregs. (**c**) Co-culture of encapsulated HCC cells and Tregs. (**d**) Immunofluorescence staining of CD133 in HCC cells before and after co-culture, and (**e**) HCC TIC (CD133 positive cells) ratio before and after co-culture, student’s t-test, * *P* < 0.05, compared with the control group. (**f**) RT-qPCR analysis of HCC TICs-related genes and (**g**) EMT-related genes, *n* = 3, student’s t-test, * *P* < 0.05, compared with the control group. (**h**) Western blot analysis of β-catenin and c-Myc in HCC cells before and after co-culture

CD4^+^ CD25^+^ CD127^−^ cells isolated from the peripheral blood of HCC patient were expanded in vitro (Fig. 1a), and nearly 93% of cells were CD4 positive, besides, among CD4^+^ cells, 96% were CD25 and FoxP3 double-positive Treg cells (Fig. 1b).

### Tregs increased the ‘stemness’ of HCC cells

After 3 days of co-culture (Fig. 1c), the ratio of CD133^+^ TICs in HCC-LM3 cells was increased significantly, from 9.856% ± 3.264–31.324% ± 6.134% (Fig. 1d-e). Besides, TICs-related genes CD133, Oct3/4, Sox2, c-Myc, Klf4, Nanog, CD13, and EpCAM were significantly upregulated (Fig. 1f). In addition, E-cadherin was down-regulated, and N-cadherin and vimentin were up-regulated in HCC-LM3 cells after co-cultured with Tregs (Fig. 1g), which indicated that Tregs induced the EMT of HCC cells to acquire TIC characteristics. Moreover, β-catenin as well as c-Myc was significantly up-regulated in HCC-LM3 cells after co-culture (Fig. 1h).

**Fig. 2:**
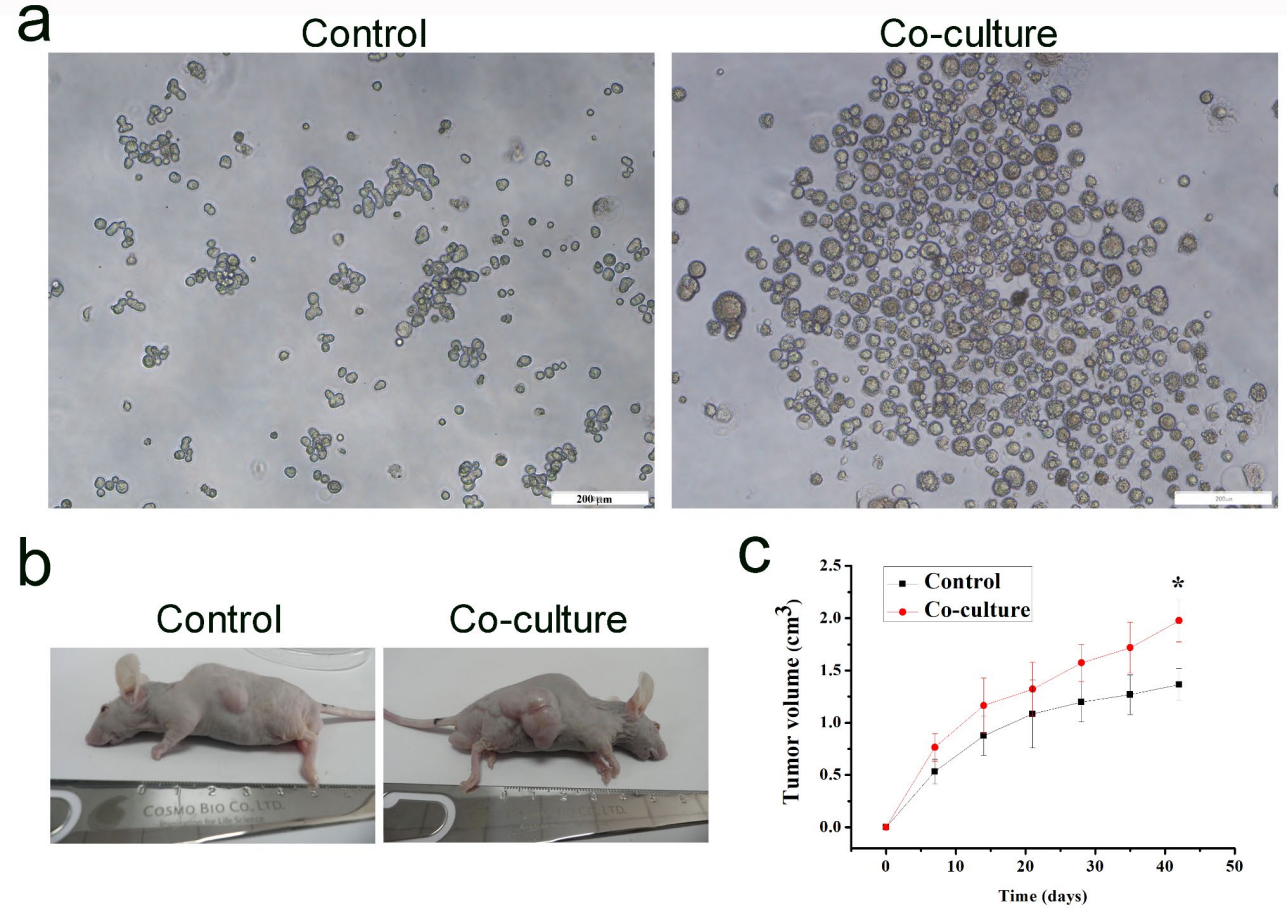
Tregs enhanced the in vitro sphere formation and in vivo tumorigenic ability of HCC-LM3 cells. (**a**) Sphere formation assay of HCC-LM3 cells before and after co-cultured with Tregs, *n* = 3, bar: 200 μm. (**b**) In vivo tumorigenicity of HCC cells before and after co-cultured with Tregs, *n* = 6. (**c**) Tumor volume change in nude mice after subcutaneously injected with 5 × 10^6^ HCC cells before and after co-culture for 6 weeks, *n* = 6, student’s t-test, * *P* < 0.05, compared with the control group

In vitro tumor sphere formation assay and in vivo tumorigenesis assay were performed to further confirm the enhancement of HCC cell ‘stemness’. After co-cultured with Tregs, HCC-LM3 cells formed more compact spheres (Fig. 2a) and also larger tumors in nude mice (Fig. 2b-c). The above-mentioned results verified that Tregs enhanced the ‘stemness’ of HCC cells.

### Tregs activated the β-catenin pathway to promote HCC ‘stemness’

**Fig. 3:**
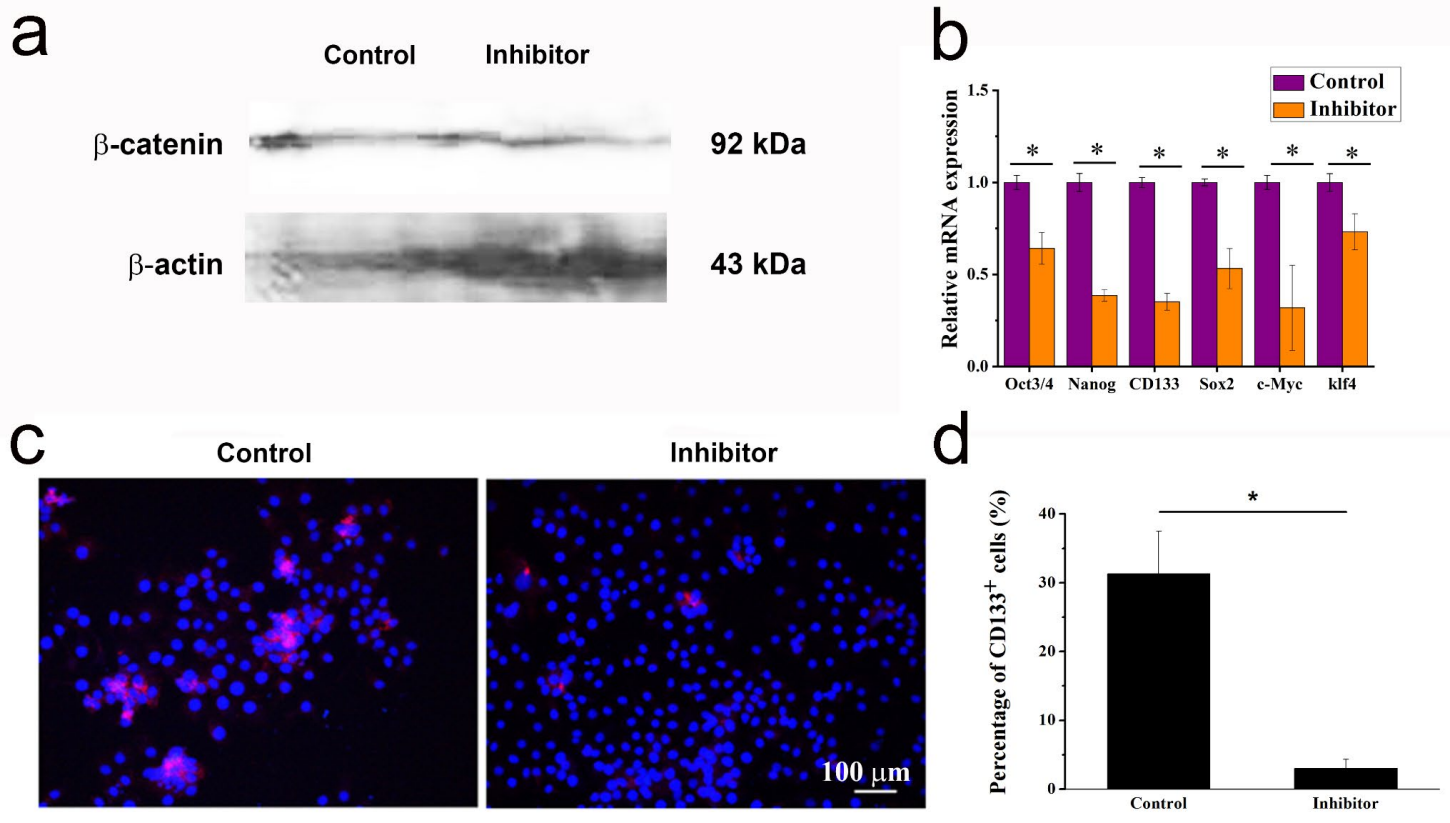
Tregs promoted TICs characteristics of HCC through the Wnt/β-catenin pathway. (**a**) Western blot analysis of β-catenin in HCC-LM3 cells with or without 5 µM XAV-939 during co-culture. (**b**) RT-qPCR analysis of TICs related genes after treatment with 5 µM XAV-939 during co-culture, *n* = 3, student’s t-test, * *P* < 0.05, compared with control group (co-culture without XAV-939). (**c**) Immunofluorescence staining of CD133 in HCC cells after treatment with 5 µM XAV-939. (**d**) Ratio of CD133^+^ TIC in HCC cells with or without 5 µM XAV-939 treatment, *n* = 3, student’s t-test, * *P* < 0.05, compared with control group

After treatment with XAV-939, Wnt/β-catenin pathway inhibitor, during co-culture, β-catenin was significantly decreased in HCC-LM3 cells (Fig. 3a), and TICs-related genes Oct3/4, Nanog, CD133, Sox2, c-Myc, Klf4 were down-regulated significantly compared to control (Fig. 3b). In addition, the ratio of CD133^+^ cells in HCC-LM3 cells was remarkably decreased, from 31.324 ± 6.134 to 3.024 ± 1.287 (Fig. 3c-d).

### Tregs inhibited FoxP3 of HCC cells to increase the TIC population

**Fig. 4:**
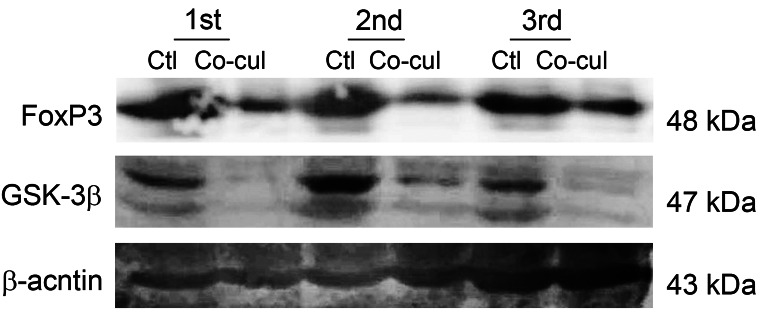
Tregs inhibited FoxP3 and GSK3β expression in HCC-LM3 cells. Western blot analysis of FoxP3, GSK3β in HCC-LM3 cells before and after co-cultured with Tregs

It was found that FoxP3 was lower expressed in HCC tissue compared to that in para-tumor tissue, in contrast, CD133 level was higher in tumor tissue (HLivH180Su10, Xinchao Biotech, Shanghai, China) (supplementary Fig. 1). After co-cultured with Tregs, FoxP3, and GSK3β were both significantly down-regulated in HCC-LM3 cells (Fig. 4).

**Fig. 5:**
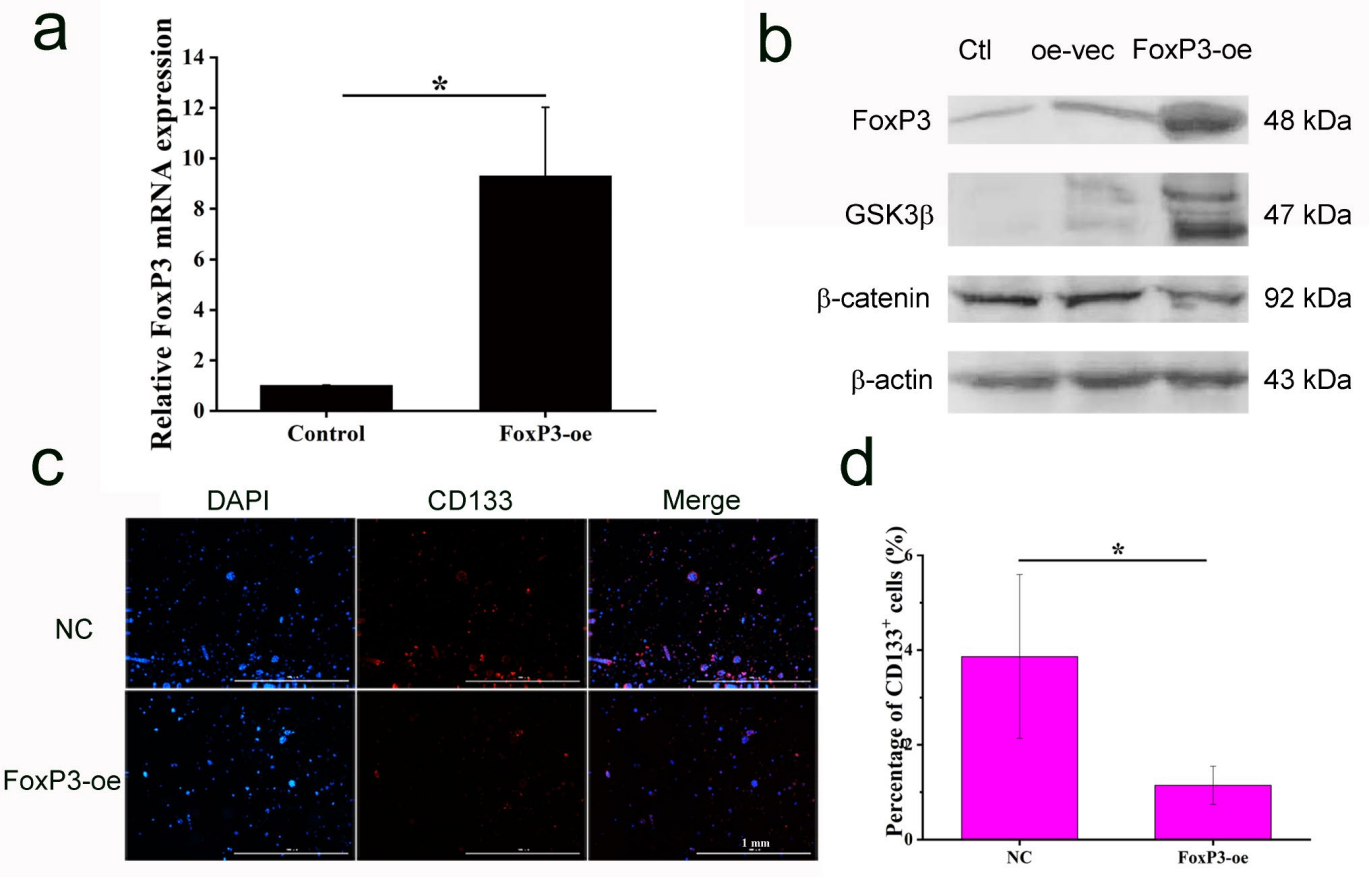
FoxP3 overexpression inhibited HCC TICs characteristics. (**a**) RT-qPCR analysis of FoxP3 mRNA expression in HCC cells after forced expression of FoxP3, *n* = 3, student’s t-test, * *P* < 0.05, compared with the control group. (**b**) Western blot analysis of FoxP3, GSK3β, and β-catenin in HCC cells after forced expression of FoxP3. (**c**) Immunofluorescence staining of CD133 in HCC cells after forced expression of FoxP3. (**d**) Ratio of CD133^+^ TIC in HCC-LM3 cells after forced expression of FoxP3, *n* = 3, student’s t-test, * *P* < 0.05, compared with the control group

Moreover, after forced expression of FoxP3 (Fig. 5a), higher expression GSK3β and lower expression of β-catenin in HCC cells were found (Fig. 5b). In addition, ratio of CD133^+^ TIC decreased from 3.863 ± 1.730 to 1.145 ± 0.423 after forced expression of FoxP3 (Fig. 5c-d).

**Fig. 6:**
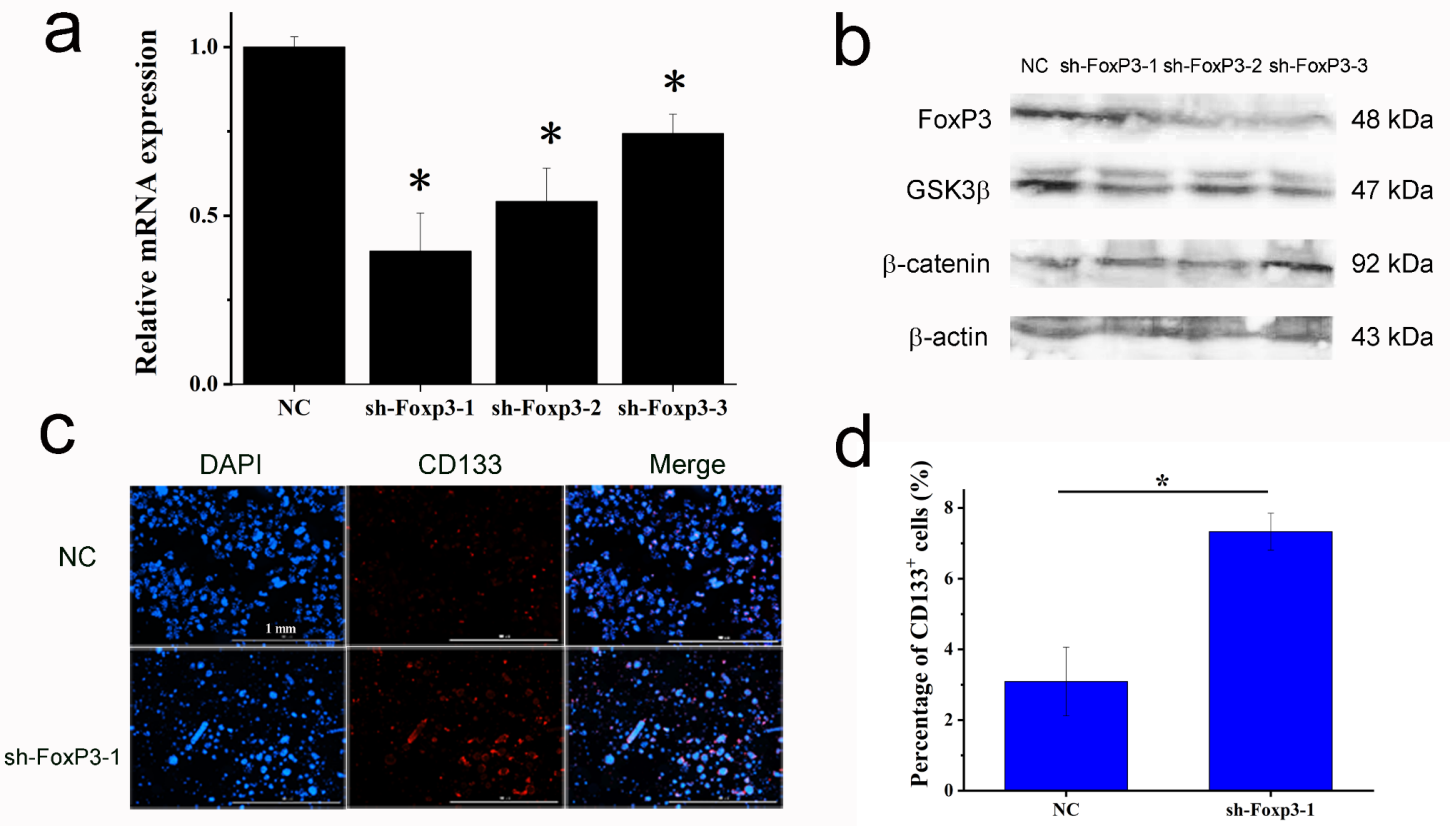
FoxP3 interference enhanced HCC cell ‘stemness’. (**a**) RT-qPCR analysis of FoxP3 mRNA in HCC-LM3 cells after three shRNA transfection, *n* = 3, student’s t-test, * *P* < 0.05, compared with control group (scramble shRNA). (**b**) Western blot analysis of FoxP3, GSK3β and β-catenin in HCC-LM3 cells after FoxP3 interference. (**c**) Immunofluorescence staining of CD133 in HCC-LM3 cells after FoxP3 interference with sh-FoxP3-1. (**d**) Ratio of CD133^+^ TIC in HCC-LM3 cells after FoxP3 interference with sh-FoxP3-1, *n* = 3, student’s t-test, * *P* < 0.05, compared with control group

Accordingly, FoxP3 interference (Fig. 6a) led to decreased GSK3β, increased β-catenin (Fig. 6b), and subsequent higher CD133^+^ TIC ratio (Fig. 6c), from 3.090 ± 0.973 to 7.329 ± 0.521 (Fig. 6d). These results suggested Tregs inhibited FoxP3 in HCC cells to increase β-catenin expression and subsequently increased the HCC TIC population.

## Discussion

Accumulating evidence indicates that therapeutic resistance and recurrence of HCC are closely associated with CSCs or TICs (Chen et al. 2015; Ishiguro et al. 2020). Nevertheless, how HCC CSCs or TICs characteristics were maintained by tumor microenvironment remains unclear.

Tregs function as dominant inhibitory components in the immune microenvironment of HCC, which are undisputed to be associated with the invasiveness of HCC, and are a promising independent predictor of recurrence and survival in HCC patients (Hassan et al. 2019; Liu et al. 2019). A few studies have reported the ability of Tregs to drive tumor cells to become TICs. Yang et al. found Foxp3^+^ IL-17^+^ Tregs induced colorectal cancer cells to up-regulated TICs-related markers including CD133, CD44s, CD166, EpCAM, and ALDH1 (Yang et al. 2011). Xu et al. disclosed that Tregs upregulated the ‘stemness’ property of breast cancer cells by increasing the side-population, promoting tumor sphere formation, and enhancing the expression of ‘stemness’-related genes including Sox2, Nanog, Oct3/4 (Xu et al. 2017).

In this study, for the first time, we found that Tregs enhanced the ‘stemness’ of HCC cells, demonstrated by increased TICs ratio, upregulated expression of TICs-related genes CD133, Oct3/4, Sox2, c-Myc, Klf4, Nanog, CD13, EpCAM, elevated tumor sphere formation, and tumorigenic ability.

FoxP3 was initially identified as a “switch” for the development and function of Tregs and thought to be restricted to hematopoietic tissues. Recently, reports have demonstrated that FoxP3 was also expressed in tumor cells, suggesting that FoxP3 might have a broader role in cancers. However, the biological function and clinical relevance of FoxP3 in tumor cells remain controversial. Some studies found that FoxP3 levels elevated in several tumor cell types, and indicated tumor progression (Grimmig et al. 2013; Merlo et al. 2009; Zeng et al. 2013). While others reported FoxP3 was a cancer suppressor gene in breast cancer (Zou et al. 2007a, 2007b), prostate cancer (Wang et al. 2009), gastric cancer (Ma et al. 2013), as well as HCC (Shi et al. 2017). Shi et al. found that higher expression of FoxP3 significantly correlated with early TNM stage, better survival, and reduced recurrence. Additionally, they demonstrated FoxP3 suppressed the proliferation and invasion of HCC cells in vitro and reduced tumor growth in vivo (Shi et al. 2017). Liu et al. reported that FoxP3 underexpression was closely related to a decreased overall survival (OS), and low FoxP3 expression was an independent risk factor for predicting OS prognosis of HCC patients (Liu et al. 2023). In this study, we found FoxP3 expression in tumor tissue was significantly lower than that in para-tumor tissue, while CD133 was significantly higher in tumor tissue compared to para-tumor tissue. Furthermore, forced expression of FoxP3 led to the significantly lower number of HCC TICs, and in contrast, FoxP3 inhibition significantly increased HCC TICs. These results were in accordance with Liu et al., which showed FoxP3 was significantly down-regulated in cancer stem cell-like cells of colorectal cancer, and forced expression of FoxP3 significantly decreased self-renewal ability of cancer stem cells including reduced side population, cancer stem cell marker CD133 expression, colonosphere formation ability in vitro, as well as tumor formation ability in vivo (Liu et al. 2017).

Abnormal initiation of the Wnt/β-catenin pathway has been recognized in HCC TICs (Guo et al. 2019). APC, Axin, CKIα, and GSK3β formed the “β-catenin destruction complex”, which connects to β­catenin molecules, phosphorylates it, and promote ubiquitylation and degradation. GSK3β functions as a switch in regulating β-catenin stability (Wu and Pan 2010). Cytoplasmic levels of β­catenin are tightly controlled by GSK3β and the degradation of β­catenin in cytoplasm could inhibit the Wnt pathway. Once GSK3β is suppressed, β-catenin accumulates in the cytoplasm and translocates into the nucleus, where it binds to the LEF/TCF complex and activates the downstream genes such as CD44, EpCAM, c-Myc, cyclin D1, among others (Vilchez et al. 2016). We found that after co-culturing with Tregs, GSK3β was significantly down-regulated and β-catenin as well as c-Myc was significantly upregulated in HCC cells. Moreover, after inhibiting β-catenin pathway, TIC-related genes along with the TIC ratio were significantly decreased.

This study, for the first time, showed Tregs enhanced the ‘stemness’ of HCC cells, nevertheless, there are some limitations. Firstly, Tregs in this study were isolated from peripheral blood, but the use of intratumoral Tregs might be closer to the real tumor microenvironment. It was demonstrated a great heterogeneity in Tregs. Tumor-infiltrating Tregs and Tregs from peripheral blood have different gene signatures. Tregs from peripheral blood showed little clonal enrichment, while much more tumor-infiltrating Tregs were clonally enriched (Zheng et al. 2017). Secondly, whether GSK3β was the direct target of FoxP3 was unrevealed in this study. Thirdly, the correlation of FoxP3 expression with the metastasis, relapse, and overall survival of HCC patients should be investigated to further confirm the tumor suppressor role of FoxP3 in HCC. Fourthly, we have evaluated if the Treg-derived exosomes played the same role as Treg cells in increasing HCC TICs (data not shown), lncRNAs (long non-coding RNAs) or miRNAs or other components in Tregs-derived exosomes responsible for increasing HCC TIC population should be identified to specify the mechanism underlying in the future studies.

## Conclusions

In summary, this study revealed Tregs increased the HCC TIC population through the suppression of FoxP3 and GSK3β and the upregulation of β-catenin. It was the first study to show that Tregs in liver tumor microenvironment regulated HCC TIC characteristics.

## Electronic supplementary material

Below is the link to the electronic supplementary material.

Supplementary Material 1

Supplementary Material 2

## Data Availability

The datasets generated during and/or analysed during the current study are available in the [figshare] repository, [10.6084/m9.figshare.25762596.v1].
