# Supplementary figures and images for: Regulatory T cells inhibit FoxP3 to increase the population of tumor initiating cells in hepatocellular carcinoma
Source: J Cancer Res Clin Oncol. 2024 Jul 29;150(7):373. doi: 10.1007/s00432-024-05892-2 (PMC11286637; doi:10.1007/s00432-024-05892-2)

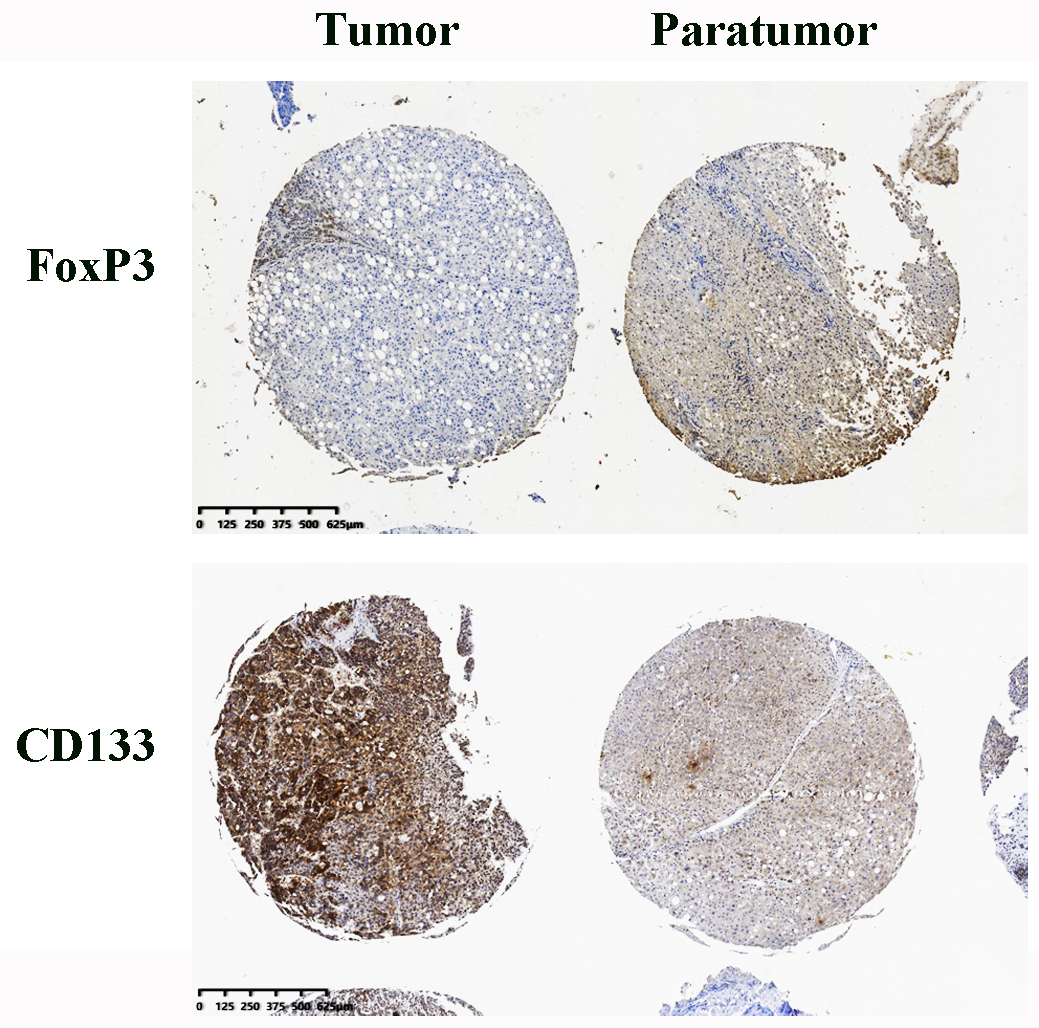

Supplement: Supplementary file 1 — Supplementary Material 1 [file 432_2024_5892_MOESM1_ESM.png]
