# Supplementary material for: Regulatory T cells inhibit FoxP3 to increase the population of tumor initiating cells in hepatocellular carcinoma
Source: J Cancer Res Clin Oncol. 2024 Jul 29;150(7):373. doi: 10.1007/s00432-024-05892-2 (PMC11286637; doi:10.1007/s00432-024-05892-2)
Supplement: Supplementary file 2 — Supplementary Material 2 [file 432_2024_5892_MOESM2_ESM.docx]

**Supplementary Table 1** Primer pairs used for quantitative real-time PCR

| **Gene** | **Sense (5’-3’)** | **Anti-sense (5’-3’)** |
| --- | --- | --- |
| GAPDH | AAGAAACCCTGGACCACCCAGC | TGGTATTCGAGAGAAGGGAGGG |
| Oct3/4 | GACAGGGGGAGGGGAGGAGCTAGG | CTTCCCTCCAACCAGTTGCCCCAAAC |
| Nanog | TCCAACATCCTGAACCTCAGCTA | AGTCGGGTTCACCAGGCATC |
| CD44 | GACGAAGACAGTCCCTGGAT | CTTCTTGACTCCCATGTGAG |
| CD133 | TGGATGCAGAACTTGACAACGT | ATACCTGCTACGACAGTCATGGT |
| CD13 | TTCAACATCACGCTTATCCACC | AGTCGAACTCACTGACAATGAAG |
| c-Myc | CACCATGCCCCTCAACGTGAACTTCACC | TTATGCACCAGAGTTTCGAAGCTGTTCG |
| Klf4 | GCGAACTCACACAGGCGAGAAACC | TCGCTTCCTCTTCCTCCGACACA |
| SOX2 | GGTTACCTCTTCCTCCCACTCCAG | TCACATGTGCGACAGGGGCAG |
| CD90 | ATGAAGGTCCTCTACTTATCCGC | GCACTGTGACGTTCTGGGA |
| FoxP3 | CACAACATGCGACCCCCTTTCACC | AGGTTGTGGCGGATGGCGTTCTTC |
